# Chimaeric antigen receptor T-cell therapy for tumour immunotherapy

**DOI:** 10.1042/BSR20160332

**Published:** 2017-01-27

**Authors:** Huan-huan Sha, Dan-dan Wang, Da-li Yan, Yong Hu, Su-jin Yang, Si-wen Liu, Ji-feng Feng

**Affiliations:** 1 Department of Chemotherapy, Jiangsu Cancer Hospital Affiliated to Nanjing Medical University, Jiangsu Institute of Cancer Research, Nanjing 210009, China; 2 Department of General Surgery, The First Clinical School of Nanjing Medical University, Nanjing 210009, China; 3 Department of General Surgery, The Fourth Clinical School of Nanjing Medical University, Nanjing 210009, China

**Keywords:** chimeric antigen receptor, immunotherapy, T cells, tumor specific or associated antigens

## Abstract

Chimaeric antigen receptor (CAR) T-cell therapies, as one of the cancer immunotherapies, have heralded a new era of treating cancer. The accumulating data, especially about CAR-modified T cells against CD19 support that CAR T-cell therapy is a highly effective immune therapy for B-cell malignancies. Apart from CD19, there have been many trials of CAR T cells directed other tumour specific or associated antigens (TSAs/TAAs) in haematologic malignancies and solid tumours. This review will briefly summarize basic CAR structure, parts of reported TSAs/TAAs, results of the clinical trials of CAR T-cell therapies as well as two life-threatening side effects. Experiments *in vivo* or *in vitro*, ongoing clinical trials and the outlook for CAR T-cell therapies also be included. Our future efforts will focus on identification of more viable cancer targets and more strategies to make CAR T-cell therapy safer.

## Introduction

Recently, cancer immunotherapies received a high degree of attention, which mainly contained the treatments for programmed death 1 (PD-1), programmed death ligand 1 (PD-1L), cytotoxic T lymphocytes-associated antigen 4 (CTLA-4) and chimaeric antigen receptors (CARs) [1]. Studies of (CAR)-specific T cells were viewed with exceptional interest for clinical development.

CAR T-cell therapy was a mode of adoptive T-cell therapy, which also contained tumour-infiltrating lymphocytes (TILs) and TCR engineered T cells (TCRT). However, TILs were reproducibly detectable only in a minority of cancers [2]. Also, the human leucocyte antigen (HLA) restricted nature of TCR recognition limited the application of TCRT to specific HLA repertoires. Fortunately, CAR T-cell therapy satisfied the need to explore new and efficacious adoptive T-cell therapy. CAR was a synthesized transmembrane protein, redirecting the target antigens expressed in tumour cells through genetic reprogramming. This gene transfer technology could efficiently introduce genes encoding CARs into immune effector cells [3]. Once transferred, engineered T cells provided specific antigens binding in a non-major histocompatibility complex (MHC) restricted manner, and were capable of recognizing tumour independently of HLA molecules. Therefore, compared with the TCRT therapy, the CARs might recognize a far greater range of potential cellular targets and could be applied to a broad range of patients irrespective of HLA phenotype [3–5]. These advantages, namely MHC-independent and tumour-specific were also carried by another approach, bi-specific T-cell engagers (BiTEs). BiTEs were a subclass of bi-specific antibodies (bsAbs). BiTEs did not need conventional MHC recognition when they induced T-cell activation through dual antigen binding. They were specific for CD3 on one arm and a tumour antigen on the second in order to bring T cells and malignant cells into close proximity [6]. However, Stone et al. [7] compared the *in vitro* sensitivity of these two strategies and found that CAR-expressing T cells were more sensitive than BiTE-treated T cells to low numbers of antigens per cell [7]. This indicated that CARs might be considered to be used in preference to BiTEs when epitope densities were low.

In decades, CAR T-cell therapy generated a great deal of enthusiasm in the field of cancer treatment. It made gratifying achievements for the treatment of haematologic malignancies like leukaemia [8] and lymphoma [9], as well as solid tumours such as neuroblastoma [10–12] and glioblastomas [13,14].

In this review, we will summarize current achievements and challenges of the CAR T-cell therapy and focus on the strategies to maximize the potential of this therapy.

## Structures, advantages and disadvantages of each generation of CAR

Over the last decades, a lot of attempts were made to construct the structures of CARs. Generally, CARs contained a targeting moiety, a transmembrane domain and an intracellular region. Specifically, a single-chain variable fragment (scFv) linked to a hinge region made up the targeting moiety, and the intracellular region was an immunoreceptor tyrosine-based activation motif (ITAM) which comprises either a region of CD3ζ chain or FcR receptor γ (FcεRIγ) [4,5].

**Figure 1:**
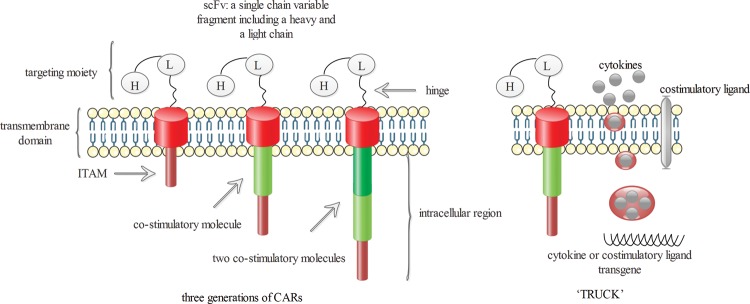
CAR T cells were classified into three generations based on intracellular signalling domains First-generation CARs contained only one signalling domain. To provide the needed co-stimulatory receptors, CD28 or 4-1BB were integrated into the second-generation CARs. Third-generation CARs had two co-stimulatory domains, typically included both CD28 and 4-1BB or CD134 (OX40). Besides this, the concept of the ‘TRUCK’ was raised. They were produced through the introduction of additional genes, including those encoding T-cell–co-stimulatory ligands (4-1BBL) or pro-inflammatory cytokines (interleukin (IL)-12).

Depending on the differences of intracellular signalling domains, CAR T cells were classified as first, second and third generation [15,16] (Figure 1). The first-generation CAR T cells just consisted of scFv and ITAM lacking co-stimulatory signalling. So, the activation and the proliferation of T cells were at a low level, leading to a short time of T-cell-killing and anti-tumour efficacy [17]. To address this limitation, the second-generation CAR T cells were designed, which expressed co-stimulatory molecules (CMs) in the intracellular domain. Concretely, they encompassed one CM such as CD28 and CD137 (4-1BB) [3,15]. The second generation showed strikingly enhanced expansion and persistence of T-cell activation, growth and survival [18]. In order to improve the efficacy, the third generation was developed based on the second generation. They had two CMs among CD28, CD27, 4-1BB and the others [3,16]. Inducted CMs into the CARs construction resulted in enhanced activation, proliferation and elevated survival of T cells so that the CAR T cells could exhibit more tumour cell-killing efficacy [16]. However, because of the presence of multiple intracellular signalling caused by the CMs of the second or third generation, an abundance of cytokines might be released and they would have resulted in cytokine storm, which was life threatening [19].

Besides, the concept of the fourth-generation CAR-modified T cells, which was also known as ‘TRUCK’ T cells, was raised by some studies [20]. The fourth-generation CAR T cells with additional genetic modification were able to express proliferative T-cell–co-stimulatory ligands (4-1BBL) or pro-inflammatory cytokines (IL-12) (Figure 1) [3]. Once recognizing the TSAs/TAAs on the tumour cells, the fourth-generation CAR T cells released a large number of perforins, granzymes and tumour necrosis factors (TNFs), which eventually led to apoptosis of tumour cells. Compared with the first three generations, the ‘TRUCK’ T cells had more advantages on affecting local suppressive cells and were enable to cause more anti-tumour destruction [21].

## TSAs/TAAs for CAR T-cell therapy

A multitude of CARs targeting an array of TSAs/TAAs have been reported for their remarkable anti-tumour effect *in vitro* or *in vivo*, including targeting cell surface tumour antigens in haematologic malignancies and solid tumours [15]. Generally, antigens recognized by CARs needed to be expressed on the surface of the tumour, making it an important disadvantage. However, interestingly, certain article showed that intracellular tumour antigens might also be recognized using the cell receptor-mimic antibody (TCRm) CAR, which derived from the ESK1 TCRm monoclonal antibody (mAb) [22]. Moreover, tumour angiogenesis was an ideal choice of the targets for CAR T-cell therapy as well [23,24]. All these possibilities made CAR T-cell therapy a powerful tool for cancer treatments.

## Cell surface tumour antigens in haematologic malignancies

### CD19

In clinical trials, CD19 was most widely used as a target tumour antigen of haematological cancers (Table 1). CD19 was considered as an ideal target for B-cell malignancies because of its high and uniform expression on B cells [25]. In 2014, Kochenderfer et al. [9] published the first report on successful treatment of diffuse large B-cell lymphoma (DLBCL), and demonstrated the feasibility and validity of anti-CD19 CAR T cells treating chemotherapy-refractory B-cell malignancies. The present study concluded 15 patients, eight achieved complete remissions (CRs). The result indicated anti-CD19 CAR T-cell therapy was a potentially powerful new treatment for B-cell malignancies. In the same year, another clinical trial showed that CR was achieved in 27 patients among a total of 30 children and adults with relapsed or refractory acute lymphoblastic leukaemia (ALL) [8]. These anti-CD19 CAR T-cell therapy overcame limitations of conventional therapies and induced remission in patients with refractory disease and the effectiveness provided strong support for further development of this approach.

**Table 1:** Published results from clinical trials of CAR T cells targeting CD19 and CD20 in haematologic malignancies

Antigens	References	Diseases	Responses to CAR T cells	Main side effects
CD19	[64]	Two FL	Two NR	None
CD19	[18]	Six NHL	Two SD, four PD	None
CD19	[75]	Eight CLL, one ALL	Two SD, one reduction in lymphadenopathy, three no objective response, one B-cell aplasia, one PD, one NE	Fever
CD19	[76]	Three CLL	Two CR, one PR	TLS, SIRS, B-cell aplasia
CD19	[19]	Three FL, four CLL, one SMZL	One CR, five PR, one PD, one NE	SIRS, B-cell aplasia
CD19	[54]	Two ALL	Two CR	SIRS, CNS toxicity
CD19	[77]	Four ALL, four CLL	Two CCR, one CR, one PR, one SD, three PD	None
CD19	[78]	Four CLL, Four MCL, two DLBCL	One CR, one PR, six SD, two PD	TLS, SIRS, fever
CD19	[53]	16 ALL	14 CR	SIRS, neurotoxicity
CD19	[9]	Nine DLBCL, four CLL, two indolent lymphomas	Eight CR, four PR, one SD, two NE	SIRS, CNS toxicity
CD19	[79]	One DLBCL, 20 ALL	14 CR, three SD, four PD	SIRS
CD19	[8]	30 relapsed and refractory ALL	27 CR, three NR	SIRS
CD19	[80]	Nine relapsed and refractory ALL	Two MRD, two CR, three PD, one CNS1, one haematological improvement and reduction in blast counts of bone marrow	CRS, neurotoxicity
CD19	[81]	14 relapsed and refractory CLL	Four CR, four PR, six NR	CRS, B-cell aplasia
CD19	[82]	One MM	One CR	B-cell aplasia
CD20	[83]	Seven FL	Two CR, one PR, four SD	Fever
CD20	[64]	Two DLBCL	Two in remission continually after autologous haematopoietic stem cell transplantation	Cytopenias
CD20	[27]	Two MCL, one FL	Two without evaluable disease remained free of progression, one PR	Fever, cytopenias
CD20	[84]	Seven DLBCL	One CR, three PR, one SD, one PD, one NE	TLS, CRS

CCR, continuous complete response; CLL, chronic lymphocytic leukaemia; CNS, central nervous system; CNS1, no detectable leukaemia in the cerebrospinal fluid; CR, complete response; CRS, cytokine release syndrome; FL, follicular lymphoma; MCL, mantle cell lymphoma; MM, multiple myeloma; MRD: minimal residual disease; NE, not evaluable; NHL, non-Hodgkin’s lymphoma; NR, no responses; PD, progressive disease; PR, partial response; SD, stable disease; SIRS, systemic inflammatory response syndrome; SMZL, splenic marginal zone lymphoma; TLS, lysis syndrome.

### CD20

CD20 (Table 1) was an activated glycosylated phosphoprotein expressed on the surface of B-lymphocytes [26]. Till et al. [27] conducted a pilot clinical trial aiming to test the effect of a third-generation CD20-specific CAR on patients with relapsed indolent B-cell and mantle cell lymphomas. The treatment was well tolerated and the clinical results of this therapy were promising with one patient having an objective partial response and two remaining free of progression for 12 and 24 months. This year, Watanabe et al. [26] concluded a research that anti-CD20 CAR T-cell therapy might also be an applicable option for the treatment of CD20-positive lymphoid malignancies. What’s more, the report found out a threshold of the antigen density. The threshold was sufficient for practical effectiveness, meanwhile, it might not result in adverse effects. Thus, antigen density seemed an ideal point for further investigation to reduce side effects.

### CD30

CD30 was a member of the TNF receptor superfamily [28]. The malignant cells in a broad variety of Hodgkin’s lymphoma (HL) and NHL selectively express CD30, which was considered as an alternative target antigen [29,30]. A phase I dose escalation study summed up that eight of nine patients with relapsed/refractory CD30 + HL or NHL treated by CAR CD30-T cells had either relapsed or progressed, showing objective anti-tumour responses [31]. However, lymphocytes and haematopoietic stem and progenitor cells (HSPCs) also expressed CD30 after activation. As for this concern, a recent research provided evidence that therapy with anti-CD30 CAR T cells derived by HRS3scFv displayed a superior therapeutic index in the treatment of CD30 + malignancies without attacking healthy activated lymphocytes and HSPCs [30]. All of these demonstrated that anti-CD30 CAR T-cell therapy could be alternative therapeutic strategy for patients with resistant/relapsed lymphomas.

### CD33

CD33 was a myeloid differentiation antigen unexpressed on pluripotent haematopoietic stem cells or inside the haematopoietic system. It could be displayed on some normal B cells, activated T cells and natural killer (NK) cells, validated as an AML target [32]. A second-generation CD33-specific CAR was generated and was proved to be effective for acute myeloid leukaemia. In the present study, leukaemia cell lines and primary tumour cells were efficiently killed *in vitro* by CAR T cells. Furthermore, the number of tumour cells was lower in mice treated with anti-CD33 CAR T cells than in control-treated mice. It showed that the anti-CD33 CAR T cells were also effective *in vivo* [33]. Therefore, anti-CD33 CAR T-cell treatment was highly effective in preventing AML development.

### CD123

CD123 was an attractive surface target highly expressed in leukaemic stem cells and leukaemic blasts but lowly expressed in normal HSPCs [34]. Mardiros et al. [35] found that their CD123 CAR T cells exhibited potent effector activity *in vitro* as well as anti-leukaemic activity *in vivo* against a xenogeneic model of disseminated AML. Another animal experiment supported that CAR T-cell therapy was a viable therapy for AML by targeting of CD123 via CAR-engineered T cells [36]. Therefore, all of these results suggested that CD123 CAR T-cell therapy was a promising immunotherapy and CD123 CAR T cells were potent candidates for future treatment of AML.

## Cell surface tumour antigens in solid tumours

### Prostate specific membrane antigen (PSMA)

PSMA was a 750-amino acid type II membrane-bound glycoprotein and abundantly expressed on the endothelium of many solid tumours, dramatically in prostate cancer [37]. A preclinical model was proposed that a second-generation anti-hPSMA CAR T cells exhibited evident and specific anti-tumour activity against a prostate tumour model, both *in vitro* and *in vivo* [38]. An experiment developed a second-generation anti-PSMA CAR T cells for improving the efficacy of first generation. The results described that the second-generation secreted more cytokines and proliferated more vigorously *in vitro* than the first generation. What’s more, the second-generation appeared to be with higher potency on suppressing prostate tumour growth in animal models [39]. Both results provided the basis for advancing such approach towards clinical application.

### Epidermal growth factor receptor variant type III (EGFRvIII)

EGFRvIII, a neo-antigen expressed in approximately 30% of glioblastomas and was correlated with poor prognosis [40]. According to Miao et al. [13], they established intracranial D-270 MG tumours, and showed that EGFRvIII CAR T cells had the capacity to suppress tumour growth and enhance survival of mice. Another report demonstrated that mice with glioma were successfully treated with a third-generation EGFRvIII CAR T cells. Significantly, the results endorsed clinical translation of this CAR in patients with brain tumours expressing EGFRvIII. [14]. Because of its highly specific and promising therapeutic efficiency, the results of clinical trials using EGFRvIII CAR T cells to treat glioblastoma attracted extensive attention.

### Disialoganglioside (GD2)

GD2 was overexpressed among paediatric and adult solid tumours, such as neuroblastoma, retinoblastoma, glioma, Ewing’s family of tumours and many other solid tumours [41]. A study tested T cells carrying the anti-GD2 CAR. The results displayed anti-cancer killing activity both in neuroblastoma cells *in vitro* and *in vivo* xenograft studies. More importantly, clinical testing of the approach was warranted in neuroblastoma and other GD2-positive malignancies due to the promising results of the study [10]. What’s more, two trails also support the anti-tumour effects of the CAR T cells specific for the GD2. One of the trails reported that four patients with neuroblastoma had evidence of tumour necrosis, including a sustained complete remission. And there were no adverse events of the therapy seen in the total 11 subjects followed for up to 24 months. The second one demonstrated three patients achieving complete remission among 11 patients with active disease of neuroblastoma and observed long-term low-level presence of CAR expressing T cells was associated with longer survival [11,12]. GD2-targeting CARs, therefore, afforded us an alternative method for treatments of neuroblastoma.

## Intracellular tumour antigens

### Wilms tumour 1 (WT1)

WT1 was overexpressed in many cancers, including haematologic malignancies, like acute and chronic leukaemias and numerous solid tumours. A study designed WT1 28z CAR T cell, the first one against a human intracellular protein, WT1. WT1 28z T cells were specific for the WT1-HLA-A*02:01 complex and the outcome provided the proof-of-concept that CAR T cells could not only target the protein expressed on the cell surface of the tumour, but also target at intracellular antigens [22].

## CAR T cells targeting tumour angiogenesis

### Vascular endothelial growth factor receptor 2 (VEGFR-2)

Tumour angiogenesis could also be a target for CAR T cells, except TSAs/TAAs. Some approaches, using VEGFR-2 CAR, aimed at targeting the tumour vasculature rather than the tumour cells because VEGFR-2 was overexpressed in tumour vasculature and was related to tumour progression and metastasis [42]. Chinnasamy et al. [23] developed a method to target tumour vasculature and the result was that the growth of five different types of established, vascularized syngeneic tumours was significantly inhibited by VEGFR-2 CAR-engineered mouse T cells plus exogenous IL-2 and the survival of mice was prolonged. Their late-stage study further displayed that co-administration of anti-VEGFR2 CAR T cells along with cells expressing a tumour-specific TCR could lead to a synergistic anti-tumour effect. Meanwhile, tumour-free survival of mice with established cancers was prolonged. All of these approaches targeting tumour angiogenesis opened new possibilities for the treatment of a wide variety of cancer types [24].

Other antigens in haematologic malignancies and in solid tumours were also well studied *in vitro* or *in vivo*, such as CD138 in MM [43], natural killer group 2 member D (NKG2D) in leukaemia and carcinoembryonic antigen (CEA) in colorectal cancer (Tables 2 and 3) [44].

**Table 2:** CAR T-cell therapies targeting other antigens in haematologic malignancies

Antigens	Diseases	*In vitro, in vivo*, in preclinical or in clinical trials	NCT ID or references
TRAIL receptor 1	Lymphoma	*In vitro*	[85]
Kappa	Lymphoma	Clinical trial	NCT00881920
CD22	FL, ALL, NHL	Clinical trial	NCT02315612
HA-1 H	Leukaemia	*In vitro*	[86]
NKG2D	Leukaemia	Clinical trial	NCT02203825
FAP	B-cell CLL	Clinical trial	NCT01722149
ROR1	CLL	Clinical trial	NCT02194374
CD138	MM	*In vitro*	[43]
	MM	Clinical trial	NCT01886976
NY-ESO-1	MM	*In vivo*	[87]
Lewis Y	MM	Clinical trial	NCT01716364

FAP, fibroblast activation protein; NY-ESO-1, New York-oesophageal-1; ROR1, receptor tyrosine kinase-like orphan receptor 1; TRAIL receptor 1, TNF-related apoptosis-inducing ligand (TRAIL) receptor 1.

**Table 3:** CAR T-cell therapies targeting other antigens in solid tumours

Antigens	Diseases	*In vitro, in vivo*, in preclinical or in clinical trials	NCT ID or references
HER2	Osteosarcoma	*In vitro*	[88]
	Breast cancer	*In vitro*	[89]
	Sarcoma	Clinical trial	NCT00902044
	Metastatic cancer	Clinical trial	NCT00924287
	Glioblastoma	Clinical trial	NCT01109095
	Solid tumours	Clinical trial	NCT01935843
CEA	Colorectal cancer	*In vivo*	[44]
	Colorectal cancer	Clinical trial	NCT00673322
	Breast cancer	Clinical trial	NCT00673829
	Liver metastases	Clinical trial	NCT01373047
	Metastatic cancers	Clinical trial	NCT01723306
CSPG4	Melanoma, breast carcinoma	*In vivo*	[90]
EphA2	Glioblastoma	*In vivo*	[91]
FR	Ovarian cancer	*In vivo*	[92]
IL-11Rα	Osteosarcoma	*In vivo*	[93]
IL-13Rα2	Glioblastoma	Preclinical trial	[94]
	Malignant blioma	Clinical trial	NCT02208362
	Refractory brain neoplasm		
	Recurrent brain neoplasm		
IL-13R	Glioma	Preclinical trial	[95]
CD171	Neuroblastoma	Clinical trial	NCT02311621
EGFR	Advanced EGFR-positive solid tumours	Clinical trial	NCT01869166
	Advanced glioma	Clinical trial	NCT02331693

CSPG-4, chondroitin sulfate proteoglycan-4; EGFR, epidermal growth factor receptor; EphA2, Eph tyrosine kinase receptor A2; FR, folate receptor; HER2, human epidermal growth factor receptor 2.

## Two serious side effects of CAR T-cell therapy and corresponding strategies

**Figure 2:**
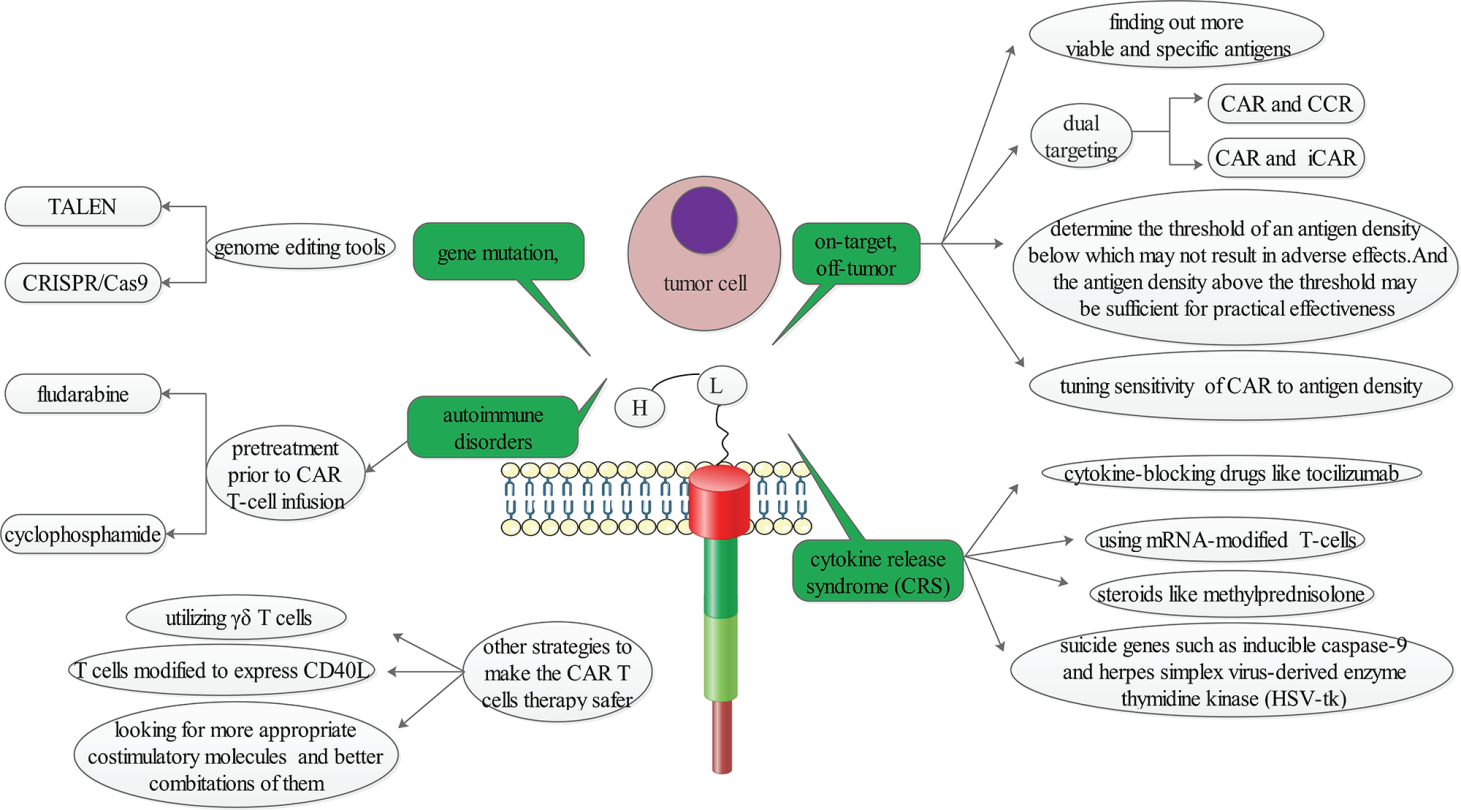
Strategies that could solve the problems including ‘on-target, off-tumour’ toxicity, ‘CRS’, ‘gene mutation’ and ‘autoimmune disorders’ and that might generate better CAR T products were taken into account

**Figure 3:**
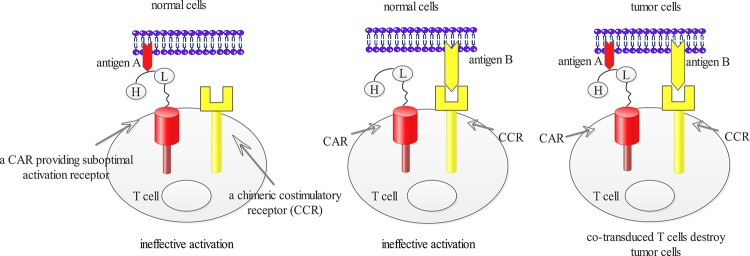
Normal cells that expressed only one antigen did not fully activate T cells T cells expressing both CCR and suboptimal activation receptor were sufficiently activated by A+B+ target cells.

**Figure 4:**
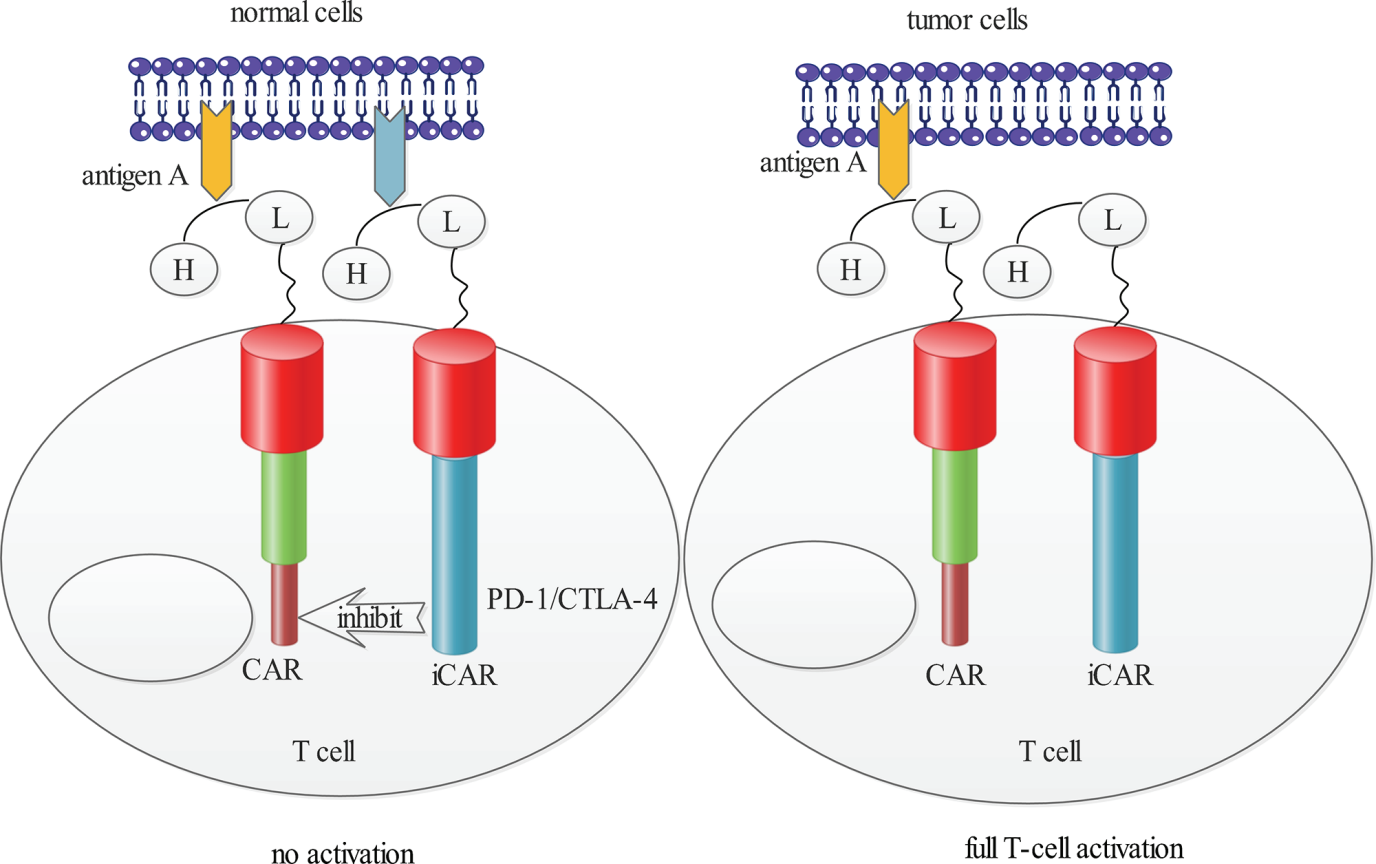
Normal cells could inhibit T-cell function when a CAR capable of full T-cell activation was co-expressed with an iCAR delivering PD-1 or CTLA-4 However, tumour cells fully activated T cells in the absence of the target for inhibitory signal.

Although positive anti-tumour effects of the CAR T-cell therapies were mentioned above, some therapies targeting other TSAs/TAAs showed obvious toxicities rather than clinical benefits (Figure 2). A case of ‘on-target, off-tumour’ toxicity was reported that one patient receiving a third generation of erbB-2-targeted CARs died on 5th day after therapy. The patient experienced respiratory distress within 15 min after cell infusion. Investigators speculated that the reason was that lung epithelial cells also expressed low level of erbB-2 recognized and damaged by CAR T cells with released mounts of cytokines [45]. In order to overcome this toxicity, it is crucial to find more specific antigens expressed on tumour cells. However, majority of the antigens also keep low density on normal tissues [46]. In considering the difficulty to find truely TSAs or TAAs, Kloss et al. [47] presented a ‘dual-targeting’ strategy. The approach transduced T cells with a CAR that provided suboptimal activation and a chimaeric co-stimulatory receptor (CCR) (Figure 3). The suboptimal activation was upon binding of one antigen whereas the CCR was used to recognize a second antigen. T cells engineered in this manner would recognize the tumour, whereas tissues expressing either antigen alone would not activate them [47]. The efficiency of T-cell activation is ineffective in the absence of simultaneous CCR recognition of the second antigen. Similarly, T cells do not react against issues only expressing the CCR because T cells would not be activated, lacking the CAR. Another alternative strategy is to employ both activating and inhibitory CAR (iCAR) that operated as logic gates (Figure 4). The iCAR delivered a dominant inhibitory signal such as PD-1 or CTLA-4 to achieve antigen-specific suppression of T-cell cytotoxicity, and the activating CAR was capable of full T-cell activation. Tumour cells expressing only the activating ligand could operate T-cell function, whereas normal cells expressing both antigens could inhibit T-cell function [48]. These approaches thereby could improve tumour selectivity.

Additionally, antigen density seemed an ideal point regarding reduction of side effects. Watanabe et al. [26] found out a threshold for antigen selection, below which might not result in adverse effects, whereas that displayed practical effectiveness above the threshold. On the other hand, the possibility of generating two CARs that differ in affinity was raised. CAR T cells in the present study were described to have the ability to distinguish malignant from normal cells based on antigen density. In the present study, EGFR-specific CAR T cells bearing low-affinity nimotuzumab-CAR selectively targeted cells overexpressing EGFR. However, they displayed diminished effector function when the density of EGFR decreased. In contrast, activation of T cells with high-affinity cetuximab-CAR showed no change [46]. Similar findings were reported including EGFRvIII-specific CAR and ROR1-specific CAR [49,50]. These methods of tuning sensitivity of CAR to antigen density made it possible to maintain anti-tumour activity without recognition of normal tissue. Many other tumours overexpressed TAAs relative to normal tissue as well. Therefore, these methods could be applied to other malignancies.

Another main safety concern was CRS. CRS was characterized by fever, hypoxia and hypotension, and potentially led to organ failure by producing several pro-inflammatory cytokines [51]. Cytokine-blocking drugs like tocilizumab that could block IL-6 signalling pathway and steroids like methylprednisolone were proved to be valid countermeasures [52–54]. Moreover, suicide genes such as inducible caspase-9 and herpes simplex virus-derived enzyme thymidine kinase (HSV-tk) were studied [55–57]. Also, the use of mRNA-modified T cells in CAR applications might be an ideal approach. A report that was the first preclinical one developed mRNA-modified CART33 cells and showed potent, but transient, *in vitro* and *in vivo* activity [58]. This solution would not permanently express CD33-specific CART cells, so it minimized the risk of long-term toxicity.

## Challenges and outlook for CAR T-cell therapy

Despite the two tough toxicities, other problems such as minimizing the risk of gene mutation, reducing autoimmune disorders and generating better CAR T products remained challenges for the CAR T-cell therapy. In order to make the CAR T-cell therapies safer, more strategies need to be considered (Figure 2).

As known, modified T cells carried the *CAR* gene, which is introduced by a retrovirus. However, the target gene is randomly inserted into the genome, so the oncogenes would be activated if the insertion site is located in the proto-oncogene area. It might lead to the high risk of gene mutation and tumorigenicity after T cells reinfused to the body. It was reported that TAL effector nuclease (TALEN) and CRISPR/Cas9 were powerful genome editing tools for safe insertion. Therapeutic transgenes could be inserted to ‘genomic safe harbours’ – chromosomal locations where transgenes would not perturb endogenous gene activity and promote cancer [59–61].

Moreover, T cells after gene modification simultaneously carried endogenous TCR and exogenous CAR, both of which might be recombinant resulting in autoimmunity [62]. Fortunately, evidence indicated that pre-treatment with fludarabine and cyclophosphamide, prior to CAR T-cell infusion was helpful to improve the efficiency of cell immunotherapy [63]. However, the study of the immunogenicity would still be in the process and of great significance for patient’s prognosis and the outcome.

In nearly all studies to date, T cells bearing αβ receptors were used [9,64]. However, αβ T cells require specific TAAs and appropriate CMs for activation. Tumour cells would be resistant to αβ T-cell-mediated cytotoxicity when there is loss of TAA expression or absence of CMs [65]. Meanwhile, success with solid tumours was limited [45,66,67]. So, generating better CAR T products seemed to be of great significance. In this regard, biological characteristics and unique functions of γδ T cells that could apply CAR T-cell therapy for solid tumours were highlighted [68]. Rischer et al. [69] suggested that γδ T cells might serve as potent and specific anti-tumour effector cells because they demonstrated that γδ CAR T cells showed cytotoxicity against tumour cell targets. Moreover, Deniger et al. [70] showed that γδ CAR T cells not only killed CD19+ tumour cell lines *in vitro*, but also inhibited tumour growth in a mouse xenograft model. More recently, another potential advantage of γδ T cells was recommended. Mirzaei et al. [68] suggested that utilizing engineered allogeneic donor-derived γδ T cells that expressed CAR transgene could be theoretically used as an off-the-shelf product because they were not restricted to MHC. All these offered the hypothesis that γδ-derived CAR T-cell product would be a promising therapeutic strategy to improve anti-tumour immune responses.

Also, Curran et al. [71] made T cells genetically modified to constitutively express CD40L with the ability to enhance T-cell proliferation and tumour cell immunogenicity. This approach not only increased CD19-specific CAR/CD40L T cells efficiency but also has profound effects on the tumour micro-environment [71]. Additionally, the incorporation of CMs used in the second- and third-generation CARs could strengthen CAR T-cell activation [3,15,16]. So, we could make efforts to find better combinations of them, which indicated that new generation of CAR T cells might be applied to cancer treatment in the near future.

## Conclusions

CAR T-cell therapy is currently perceived as one of the most promising therapeutic approaches for cancer treatment because of significant outcomes in various studies [5,72]. Instead of traditional drug and radiation therapy, CAR T-cell therapy may reduce or even replace some bone marrow transplantations for haematologic malignancies, avoiding high cost of long-term hospitalization and high risk of bone marrow transplantations. However, the application of CAR T-cell therapy still requires efforts and multiple exploratory studies to limit or contain the side effects. Furthermore, cell preparation, functional testing, evaluation of therapeutic efficacy and so on are also required systematic knowledge and norms.

Additionally, some experiments demonstrated that CAR-transduced cytokine-induced killer (CIK) and NK cells were effective [73,74]. To assess the validity, the safety of these approaches appears to be important and necessary. So, we can expect that CIK and NK cells may also be well involved in more clinical CAR therapies in the future.

We believe that with the growing understanding of technology foundations and clinical researches, CAR T-cell therapy will have a promising role in tumour immunotherapy.

## Abbreviations

ALL: acute lymphoblastic leukaemia
AML: acute myeloblastic leukaemia
BiTE: bi-specific T-cell engager
CAR: chimaeric antigen receptor
CEA: carcinoembryonic antigen
CLL: chronic lymphocytic leukaemia
CM: co-stimulatory molecule
CTLA-4: cytotoxic T lymphocytes-associated antigen 4
EGFR: epidermal growth factor receptor
HL: Hodgkin’s lymphoma
HLA: human leucocyte antigen
HSPC: haematopoietic stem and progenitor cell
iCAR: inhibitory CAR
IL: interleukin
ITAM: immunoreceptor tyrosine-based activation motif
MCL: mantle cell lymphoma
MHC: major histocompatibility complex
MM: multiple myeloma
NHL: non-Hodgkin’s lymphoma
NKG2D: natural killer group 2 member D
NK: natural killer
PSMA: prostate specific membrane antigen
ROR1: receptor tyrosine kinase-like orphan receptor 1
TALEN: TAL effector nuclease
TCR: T cell receptor
TCRT: TCR engineered T cells
TIL: tumour-infiltrating lymphocyte
TNF: tumour necrosis factor
TSA: tumour specific antigens
TSS: tumour associated antigens
